# Valuable Cholera Prescription

**Published:** 1902-12

**Authors:** N. F. Howard

**Affiliations:** Dahlonega, Ga.


### CORRESPONDENCE.

### VALUABLE CHOLERA PRESCRIPTION.

*Editor Atlanta Journal-Record of Medicine:*

| | |
|---|---|
| Syrup rhei comp | ℥ ii |
| Tincture opium | ℥ i |
| Ess. peppermint | ℥ i |
| Tincture ginger | ℥ i |
| Spirits lavender comp | ℥ ii |
| Gum camphor | ʒ ii |

Mix.

Dose:—10 drops to a teaspoonful according to age of patient, in a small quantity of water every 1, 2 or 3 hours, as may be needed for cholera, cholera morbus, cholera infantum, sick stomach, diarrhea and colic. Dose for infants and children up to 9 years of age, give one drop for each year of its age.

We have used this "Cholera Mixture" for twenty-five years or more, with better results in the relief of these complaints than any other remedy.

And we have also found its use in the acute forms of dysentery very efficient in the relief of the pains accompanying that disease.

N. F. Howard, M.D.,

November 21, 1902. Dahlonega, Ga.

